# Encoding Odorant Identity by Spiking Packets of Rate-Invariant Neurons in Awake Mice

**DOI:** 10.1371/journal.pone.0030155

**Published:** 2012-01-17

**Authors:** Olivier Gschwend, Jonathan Beroud, Alan Carleton

**Affiliations:** 1 Department of Basic Neurosciences, School of Medicine, University of Geneva, Geneva, Switzerland; 2 Geneva Neuroscience Center, University of Geneva, Geneva, Switzerland; Center for Genomic Regulation, Spain

## Abstract

**Background:**

How do neural networks encode sensory information? Following sensory stimulation, neural coding is commonly assumed to be based on neurons changing their firing rate. In contrast, both theoretical works and experiments in several sensory systems showed that neurons could encode information as coordinated cell assemblies by adjusting their spike timing and without changing their firing rate. Nevertheless, in the olfactory system, there is little experimental evidence supporting such model.

**Methodology/Principal Findings:**

To study these issues, we implanted tetrodes in the olfactory bulb of awake mice to record the odorant-evoked activity of mitral/tufted (M/T) cells. We showed that following odorant presentation, most M/T neurons do not significantly change their firing rate over a breathing cycle but rather respond to odorant stimulation by redistributing their firing activity within respiratory cycles. In addition, we showed that sensory information can be encoded by cell assemblies composed of such neurons, thus supporting the idea that coordinated populations of globally rate-invariant neurons could be efficiently used to convey information about the odorant identity. We showed that different coding schemes can convey high amount of odorant information for specific read-out time window. Finally we showed that the optimal readout time window corresponds to the duration of gamma oscillations cycles.

**Conclusion:**

We propose that odorant can be encoded by population of cells that exhibit fine temporal tuning of spiking activity while displaying weak or no firing rate change. These cell assemblies may transfer sensory information in spiking packets sequence using the gamma oscillations as a clock. This would allow the system to reach a tradeoff between rapid and accurate odorant discrimination.

## Introduction

Sensory perception is driven in the brain by specific coding strategies defined as modification of firing patterns in particular subpopulations of neurons. The rate code is the most studied as it implies firing changes in neurons which are experimentally easy to detect and quantify. However, other codes are also considered since they may carry complementary information and/or may be more resistant to noise or fluctuations of the stimulus. These include, temporal codes such as synchronized firing of neurons [1], [2], [3], [4], [5], [6], [7], first spike latency following stimulus onset [8], [9], [10], [11], [12] or firing in a specific phase of particular rhythms [13], [14], [15], [16]. Several codes may also be multiplexed in order to increase the total amount of information embedded in neural responses [17], [18], [19].

Neuron responsiveness is assessed at a single cell level, usually focusing on rate changes relative to baseline. In anesthetized mammals, mitral/tufted (M/T) cells in the olfactory bulb (OB) respond to odorants by exhibiting large changes in their firing rate [20], [21], [22], [23], [24], [25]. Moreover, most M/T cells respond to only few odorants, leading to the conclusion of sparse coding in the OB [21], [23], [25]. On the other hand, M/T cells have been reported to respond to odorants by finer temporal changes of rate, such as tuning in the first spike latency or the preferred phase of discharge in the respiratory cycle [22], [26], [27], [28], [29].

Theoretical studies showed that populations of rate-invariant neurons may encode information as cell assemblies driven by a common oscillation [2], [30], [31]. While few studies in rodents have shown that M/T cells encode information in specific time-windows [22], [32], none of them emphasized that the information can be caried by rate-invariant cells changing their temporal patterning. In addition, to our knowledge, no experimental evidence supports the potential impact of physiological oscillations, such as gamma oscillations, in driving M/T cells activity and conveying odorant information.

To address these issues, we have recorded populations of M/T cells in anesthetized and in awake mice. Upon comparing odorant evoked activity to baseline activity, we found that, over the complete breathing cycle, most neurons in awake animals are rate invariant. Moreover, this neuronal population expresses fine temporal changes in firing inside the respiration cycle that contain sufficient information to be used to discriminate between different odorants. We have tested, on a single trial basis, the robustness of different codes at different time scales to extract stimulus information from a neuronal population. Our data suggest that in order to reach fast and accurate odorant discrimination, the odorant information has to be conveyed as spiking packets read over gamma oscillations used as an internal clock.

## Materials and Methods

### Animal preparation

All experiments were performed on 8 to 16 weeks old male C57BL/6J mice (Charles River France) and were in accordance with the Swiss Federal Act on Animal Protection and Swiss Animal Protection Ordinance. Our experiments were approved by the university of Geneva and Geneva state ethics committees (authorization 1007/3387/2).

For the anesthetized recordings, mice were prepared as described previously [22]. For the awake recordings, mice were anesthetized with isoflurane (3–4% induction, 1–2% maintenance). The skin overlaying the skull was removed under local anesthesia using carbostesin (AstraZeneca, Zug, Switzerland). A metallic head post was then fixed on the bone by embedding its base in dental cement (Omni-Etch Dentin, OmniDent). The rest of the skull was also covered with dental cement except the part overlaying the OB. We did not notice any behavioral changes due to the head post fixation after placing back the mice in their home cage. The animals were exploring, cleaning themselves and presenting a level of activity similar to the normal conditions. They did not present any pain related behavior such as hunched posture, social isolation or body shaking.

Few days after recovery, a mouse was placed in a plastic tube and head-fixed by screwing the head post on a custom made metalic device fixed on the air table. The mice were trained to this restraining condition for 2–4 sessions (30–60 min each) done in 2 days. The day of the experiment, a mouse is head restrained and anesthetized using isoflurane (3% induction, 0.75–1% maintainance; by placing in front of the snout a tube delivering the anesthetic) in order to do a craniotomy on top of the olfactory bulb. At the end of the craniotomy, the tetrodes are placed at the surface of the olfactory bulb, the entire procedure lasting ∼15–30 min. Then we penetrate the electrodes into the bulb and we look for the M/T neurons and once found, we leave the electrodes in place and stop the anesthesia. The experimental protocol and recordings started around 45–60 min after complete recovery of the animal.

For all experiments, respiration was monitored using a directional air flow sensor (AWM2100V, Honeywell, MN) placed in front of the mouse nose. We observed an average breathing cycle period of 393 ms in awake animals (15.8 ms inter animal S.D; *n = *6) and 352 ms in anesthetized animals (12.8 ms inter animal S.D; *n = *12). The device, though close to the snout, does not prevent the odorant to reach the animal nostril.

### Odor delivery and experimental protocol

All odorants (amyl acetate: Aa, ethyl butyrate: Eb, hexanone: Heb) were from Sigma-Aldrich. As odorant stimuli, we used the following mixtures: amyl acetate/air 60%/40%, ethyl butyrate/air 60%/40%, 3-hexanone 60%/40%, amyl acetate/ethyl butyrate 60%/40% and 40%/60%, 3-hexanone/ethyl butyrate 60%/40% and 40%/60%. Each stimulus was repeated 9 and 5 times for anesthetized and awake mice datasets respectively. To test the impact of the number of repetitions for each stimulus (see below), we acquired another dataset and used 8 different stimuli, each applied individually 20 times (Fig. S1). All are monomolecular odorants evoking different percept, at least in Humans: amyl acetate, methyl benzoate, ethyl butyrate, geraniol, carvone−(+), carvone−(−), octanal, 3-hexanone.

Four milliliters of pure monomolecular odorant were placed in glass vials. Odorants were delivered for 2 seconds through a custom made olfactometer as described previously [22], [57]. The odorant onset was set to arrive during an animal's expiration. An air flow passed through the vials containing the odorants and was further diluted 20 times with clean dry air before being sent to the nose. All mixtures were performed by gas mixing, varying the relative flow of independent stream of odorized air. Because odors were delivered ∼1 cm away from the animal's nose, these values overestimate concentrations actually reaching the nasal cavity. The total flow was constant (0.4 l/min). To maintain a stable odor concentration during the entire stimulus application, we ensured that flows were stationary with a 5 s preloading before the odorant was delivered.

### *In vivo* electrophysiological recordings and spike sorting

A 1–2 mm window was drilled above the olfactory bulb and dura mater was opened. One or two silicon-based recording electrodes (A-4×2-Tet-5 mm-150-200-312, NeuroNexus Technologies, Ann Arbor, MI, USA) were inserted. The skull cavity was filled with a mixture of wax and paraffin or an ophthalmic gel (Lacryvisc, Alcon) to protect the brain from drying. During recordings of awake mice, a silver wire contacting the gel was connected to the air table to ground the preparation. Electrodes were lowered vertically in the target zone until the dorsal or medial mitral/tufted cell layer (MCL) was reached. The M/T cell layer was clearly recognised by its strong extracellular spiking activity restricted to a 100–150 um depth variation. This contrasted with the much less prominent spiking activity in the external plexiform and the granule cell layer [39], [47], [58]. In this respect, it is noteworthy that electrodes we used had low impedances (1 to 4 MΩ at 1 kHz). The conditions for optimal single-cell identification are stability and reasonable size of the extracellular spike with respect to background noise (clustering, see below), which in the case of low impedance electrodes could only be fulfilled by MCs and tufted cells (the larger cells in the OB), as observed by others in mice [47] and in rat [39], [58]. As a confirmation, we detected almost no clusterable activity in the granule cell layer, which contains a large density of small neurons.

Wide-band field potentials were amplified (100×) and band-pass filtered (0.1 Hz to 9 kHz). All data was digitized at 32556 Hz with the Cheetah Digital Lynx system (Neuralynx, Tucson, AZ). Further details about recording and spike sorting have been described extensively elsewhere [22].

Individual neurons were finally identified as the clusters showing a clean refractory period in their autocorrelograms. A total of 102 and 130 isolated neurons were recorded in 12 anesthetized and 6 awake mice respectively. The number of cells recorded per animal ranged between 1 to 25 cells. For the experiments testing the dependence of the codes to the number of odorant repetitions, we recorded 46 neurons in 5 mice. All subsequent analyses and statistics were calculated using custom scripts written for Matlab (MathWorks, Inc., Natick, MA) or C.

### Data analysis

#### Breathing cycle realignment

Respiratory cycles (RC) durations of awake mice were highly variable within and across trials. In order to analyze the consistent neural responses to odors across trials, the beginning of each cycles were temporally realigned to each other. All RC were artificially matched to the average breathing duration (393±15.8 S.D.) over the 5 trials: longer cycles were cut and shorter ones were prolonged. Corresponding spiking period were realigned with the same method. Importantly, relative action potential timings in spike trains were not affected by this method (Fig. S1A–B).

#### Statistical analysis of the rate change for single cell responses

Change in the average respiratory cycle firing rate during odor presentation relative to baseline was assessed by the non-parametric Wilcoxon rank sum test repeated in each respiratory cycles spanning stimulus presentation and for all 7 stimuli. In a particular cycle, a cell was considered as responsive if at least one odorant stimulus evoked a significant change in firing in comparison to baseline. We set the α-value to 0.05 and further applied a Bonferroni correction for multiple testing (i.e. division by stimulus number: 7) so that all resulting percentages could not be overestimated by more than 5% of false positive.

#### Analysis of the tuning in spike timing

We divided each respiratory cycle in 8 time bins (on average, anesthetized: 43 ms, awake: 49 ms) in which we computed the average firing rate. For each trial, we described the firing activity per trial over consecutive respiratory cycles by a 8× *n* matrix (*n* respiratory cycles before and during odor presentation). For each odor, we further averaged all matrices computed for individual trials. The same process was applied for all the 7 odors and the averaged matrices were concatenated together (total size, 8×7*n*). A principal component analysis (PCA) was computed with the concatanated matrix, which allowed representing all the RC as a vector in a multidimensional space of 8 dimensions, each of them representing one of the respiratory bins. The 3 first dimensions of the PCA transformation carried more than ∼75% of the variance. To define if neurons are responding to odors, we assessed whether firing distribution in RC of the baseline and odor periods are significantly different. For that, in the PCA space, we measured the Euclidean distances between RC of the odor and baseline periods.

**Figure 1:**
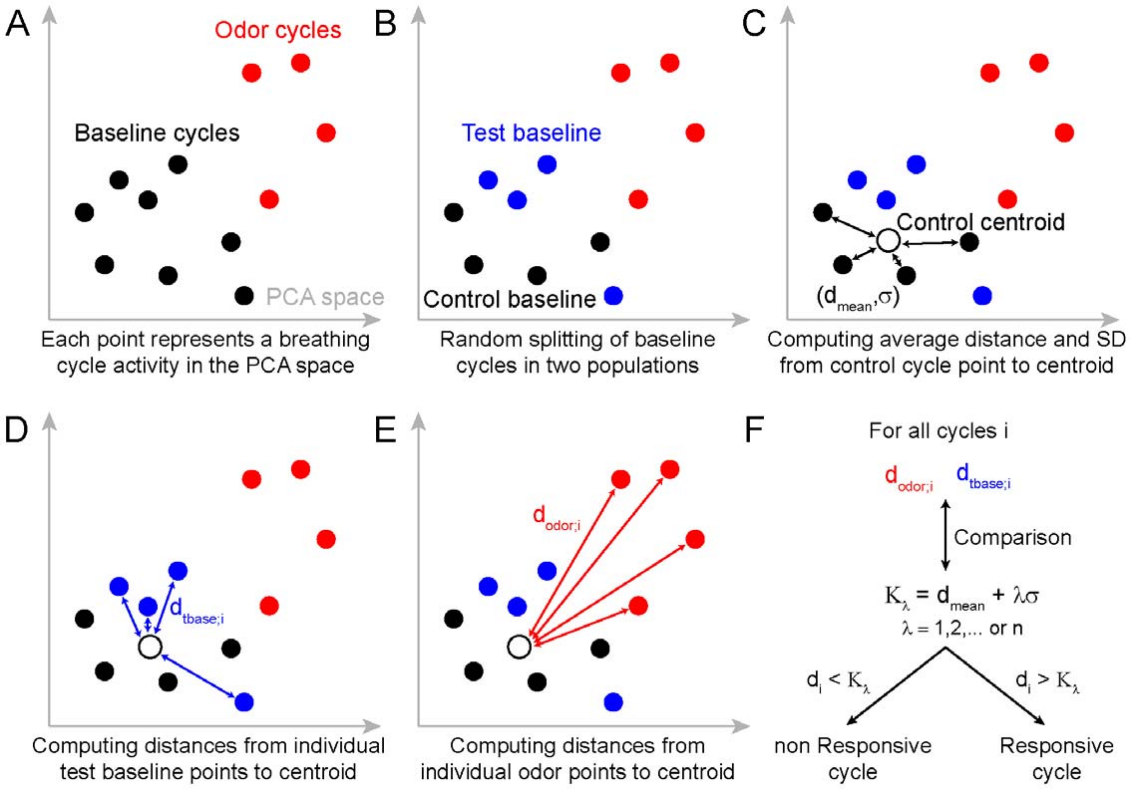
Framework used to detect for individual neurons fine changes in firing mediated by odorant presentation. (**A**) Each breathing cycle spiking activity is described by a vector of 8 dimensions. Following a principal component analysis (PCA) on all baseline and odor cycle vectors, each cycle point is plotted in the PCA space. (**B**) The baseline points cloud is randomly split in two sets of points further referred as control and test baseline. (**C**) The centroid of the control baseline is calculated and all individual baseline points to centroid distances are computed. From that, we extract the average point to centroid distance (d_mean_) and standard deviation (σ). (**D**) Distances (d_tbase;i_) between test baseline cycle i and centroid are computed. (**E**) Distances (d_odor;i_) between odor cycle i and centroid are computed. (**F**) A cycle activity is considered to be different from the test baseline activity if the distance d_i_ is superior to K_λ_ the summation of the average distance (d_mean_) and λ standard deviation, in which λ is parametrically varied.

First, the RCs were segregated into baseline respiratory cycles (BRCs) and odor-evoked respiratory cycles (ORCs) (Fig. 1A). The BRC was again divided randomly into two groups with an equivalent number of BRC: the control BRC (CBRC) and the test BRC (TBRC) (Fig. 1B). We then computed the CBRC centroid and the average distance (d_mean_) and the standard deviation (σ) of the Euclidean distances between the CBRC centroid and each of the single CBRC (Fig. 1C). Then the distances (K_o_) between each single OBRC and the CBRC centroid were calculated (Fig. 1E). A cell was considered as responsive if:
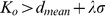
(1)where λ = 1, 2, 3 … n, for at least one odor. However, this method may detect false-positive response. In order to discard them, we computed in a same manner the distances (K_b_) between TBRC and the CBRC centroid (Fig. 1D). Similarly, if:
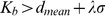
(2)a cell was considered as responsive for at least one odor (where λ = 1, 2, 3 … n). The number λ of standard deviation σ was parametrically increased until only 5% of cells were considered as responsive using the CBRC template. We thus kept the value of λ extracted from the equation (2) and used it to satisfy the conditions in the equation (1). This led to a detection of responsive cell with a risk of false-positive inferior or equal to 5%.

In this analysis, the cell had to respond significantly for at least 3 RC to be considered as responsive. Similar results were observed by considering one or two significant RC but the exact percentages of responsive cells were lower due to an increase of false positive in the baseline.

#### Population vector construction and prediction algorithm

**Figure 2:**
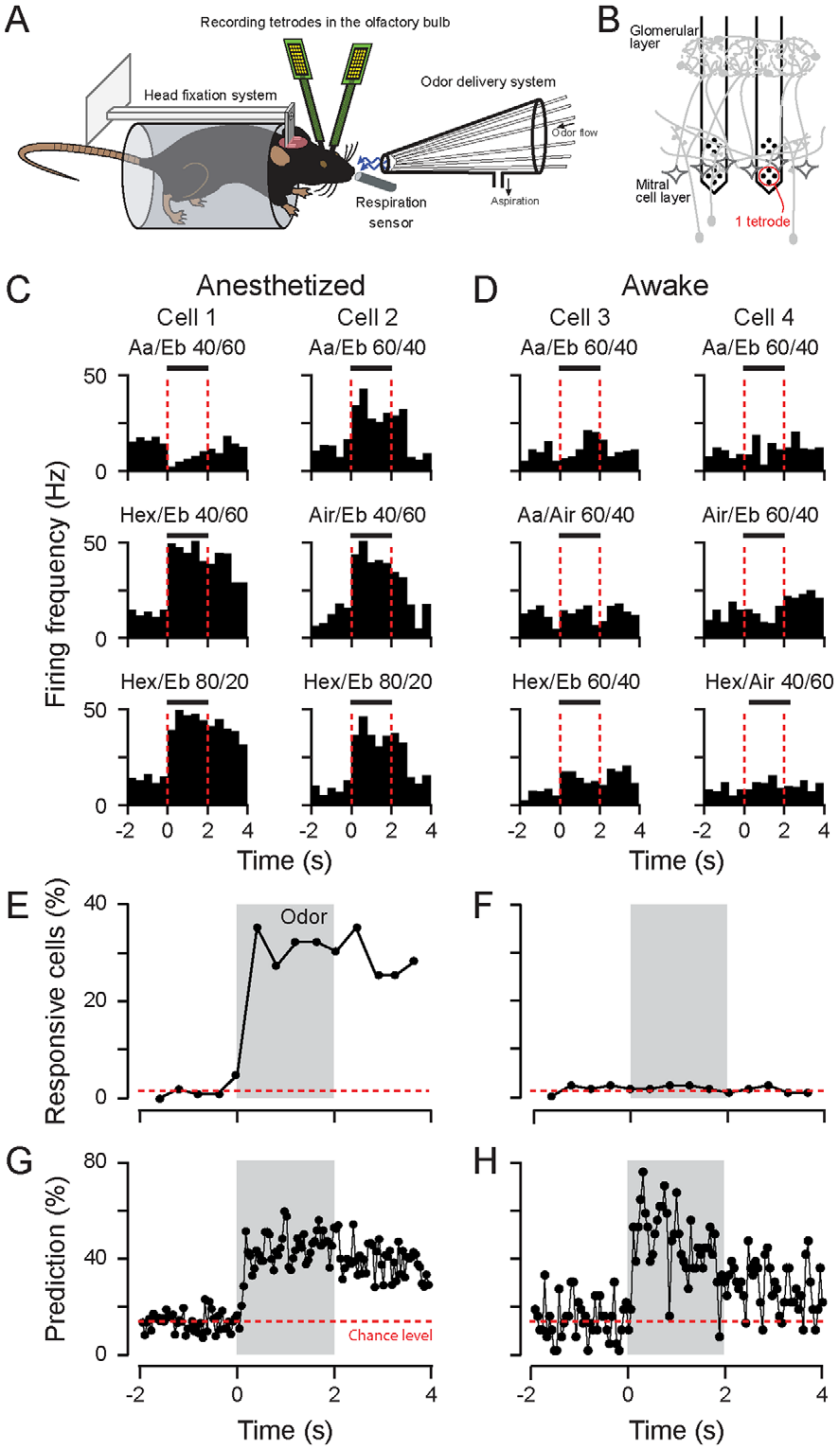
Rate-invariant mitral/tufted cells encode odorants in awake mice. (**A**) Sketch of the experimental setup. The mice are head-restrained and tetrodes are placed in their olfactory bulb. The repiratory cycles are measured by a sensor placed in front of the animal nose. (**B**) Schematic positioning of the electrodes during tetrode recordings. The electrodes were lowered from the olfactory bulb surface until the typical activity of the mitral/tufted (M/T) cell layer was observed. (**C–D**) Representative examples of M/T cells responses to selected odors shown as a peristimulus time histogram (PSTH) in anesthetized (**C**) and awake (**D**) mice. The time bin is set to the breathing cycle duration. The vertical dashed red lines define the odor application period. The odors are mixtures of amyl acetate (Aa), ethyl butyrate (Eb) and 3-hexanone (Hex) at different ratios. (**E–F**) Percentage of M/T cells that significantly changed their firing rate to at least one (of 7) odorant in anesthetized (**E**) and awake (**F**) mice. Grey boxes indicate odorant applications. Horizontal red dotted line indicates the level of false responsive cells computed over the baseline. (**G–H**) Correct stimulus decoding prediction based on population activity computed on single trials. The breathing cycles are divided in eight time bins. Red bars: chance level. Population of neurons: 102 and 130 cells for anesthetized (**E,G**) and awake (**F,H**), respectively.

**Figure 3:**
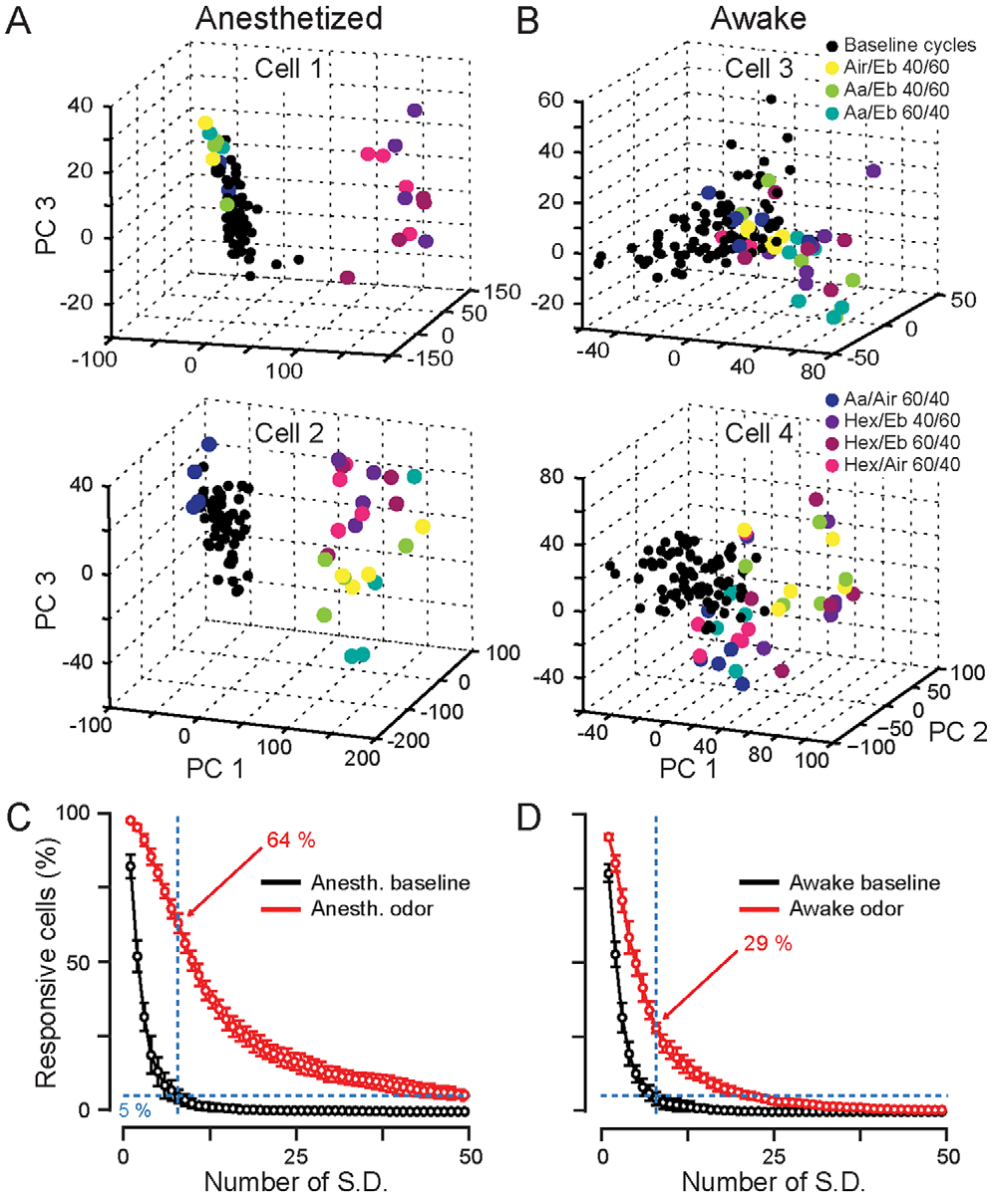
Reorganization of spike timing during respiratory cycle discharge in individual neurons following odorant presentation in awake mice. (**A–B**) Examples of four mitral/tufted cells, recorded in anesthetized (**A**) and awake (**B**) animals, showing changes in respiratory cycles firing during odorant application. Each point represents a breathing cycle activity (BC). Black points: BCs during baseline. Colored points: BCs during odorant application. The color code corresponds to the different stimuli. Note that colored BCs located in the baseline cloud correspond to non-responsive odorants. (**C–D**) Percentage of the recorded cells responding to at least one odorant in anesthetized (**C**) and awake (**D**) mice. A cell is considered as responsive when a significant difference between baseline and odor cycles firing is found using a method that takes into account both rate and temporal changes (see Methods and Fig. 1). We determine if an odorant BC is different from the baseline BC cloud taking into consideration the variance of the baseline cloud (standard deviation, S.D.). We use a test set of baseline cycles to estimate the percentage of false positives and varied the number of S.D. necessary to reach a maximum of 5% of them (horizontal blue dotted lines). With such quantifications, 64% and 29% of the neurons in anesthetized and awake mice (vertical blue dotted lines), respectively, are found to respond to at least one odorant.

**Figure 4:**
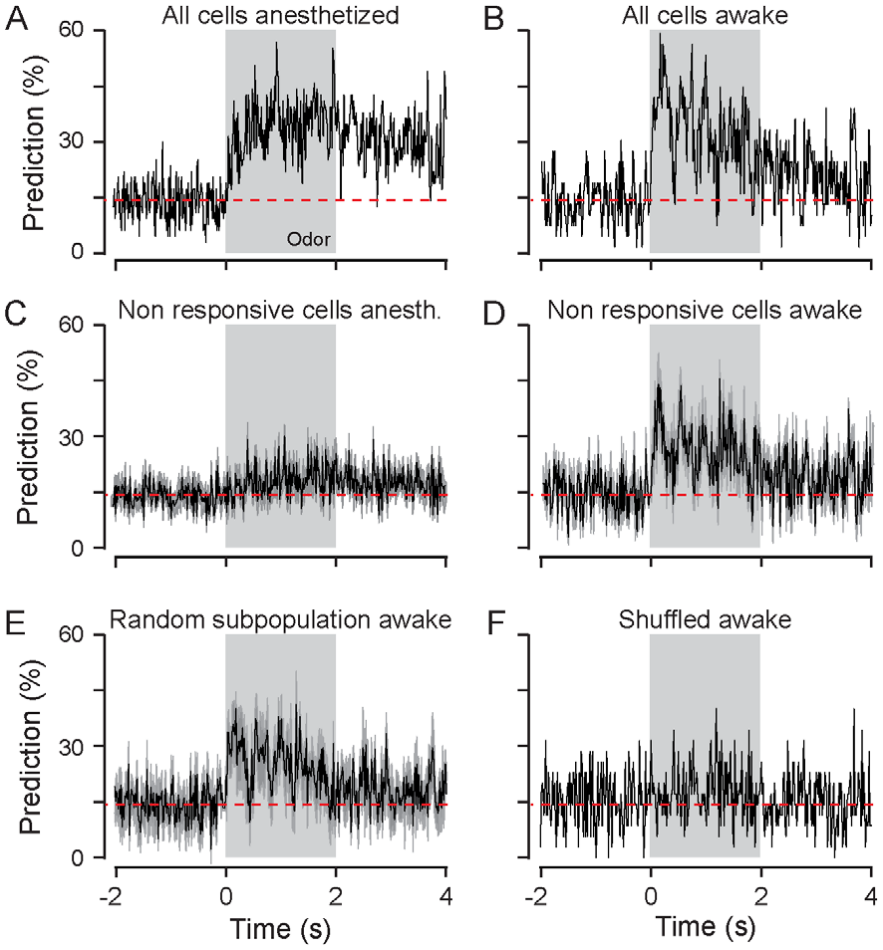
Populations of rate-invariant neurons encode olfactory information in awake mice. (**A**) Correct stimulus prediction curve computed over time for odor identity using 102 cells recorded in anesthetized mice. Breathing cycles were divided in 24 time bins (duration: 14 ms – to reflect gamma frequency). Grey box: odor application. Red line: chance level. (**B**) Correct prediction curve computed with a population of 130 cells recorded in awake mice. Time bins duration: 16 ms. (**C–D**) Prediction performance calculated only using non-responsive cells identified with the analysis presented in Figure 2 in anesthetized mice (**C**, *n = *37 cells on average) and in awake mice (**D**, *n = *92 cells on average). (**E**) Correct prediction curve using randomly selected subpopulation of cells in awake in order to match the same number of cells as in (**C**). (**F**) Shuffling spike trains independently for each cell removed all information contained in the entire ensemble (130 cells).

**Figure 5:**
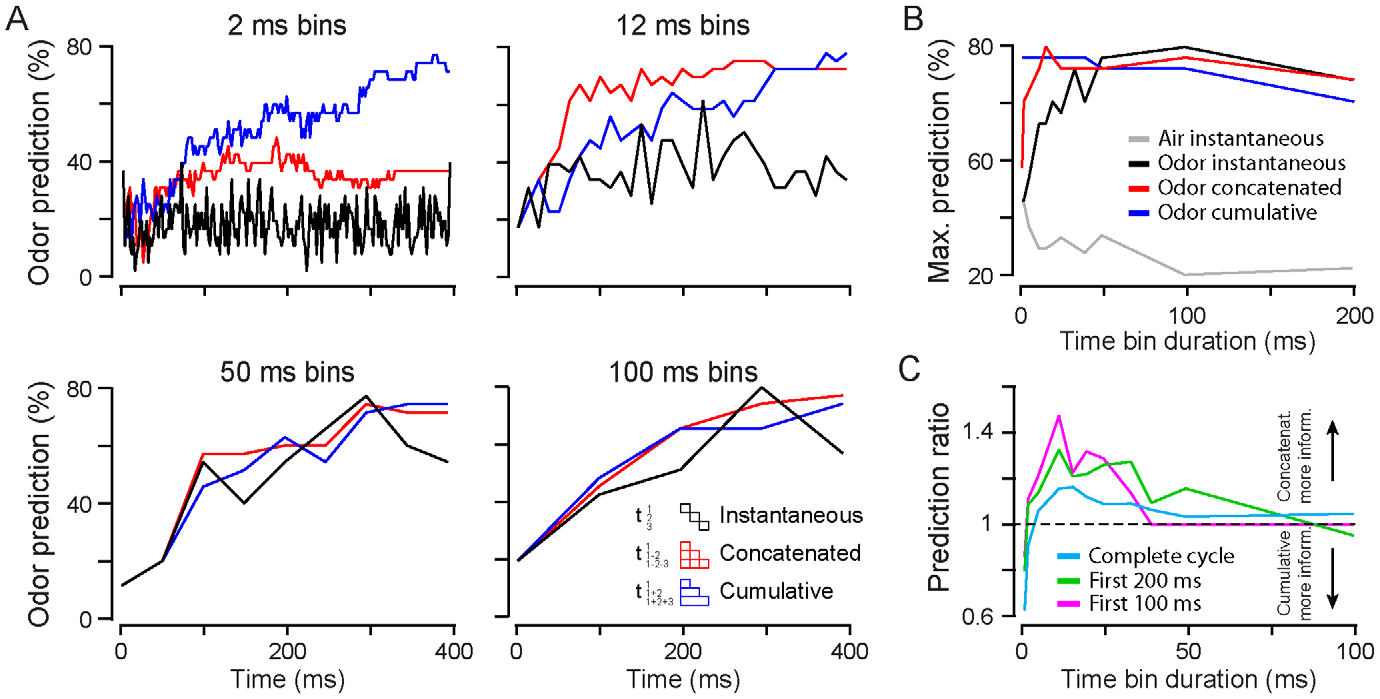
Impact of the reading time window duration on different decoding mechanisms. (**A**) Correct stimulus prediction curves during the first breathing cycle (during odorant application) computed for different coding schemes and different time window durations. *Black curves*: instantaneous firing rate (i.e. in independent time bins). *Red curves*: concatenated firing over consecutive time bins (temporal sequence analysis). *Blue curves*: cumulative spike counts over consecutive time bins. Each graph shows the prediction curves computed with a population vector built with different time bin durations (2, 12, 50 and 100 ms). (**B**) Maximum of prediction in the first breathing cycle computed for different bin durations and for the different coding schemes during odorant presentations. Noisy fluctuations of the prediction curves increase for analysis using short bin duration, leading to an overestimation of the true maximum of prediction. For comparison, the maximum of correct prediction computed on a respiratory cycle before odorant application is plotted. (**C**) Ratio of the prediction curves for the concatenated code over the cumulative code. For each window duration, a ratio between the prediction curves of the concatenated and cumulative schemes is computed either on the first 100 ms (*magenta*), 200 ms (*green*) after odor onset (i.e. for 0 to 100 or 200 respectively) or the entire respiratory cycle duration (*cyan*). Cumulative code is more informative for shorter time bin duration (<4–6 ms) while concatenated code becomes more informative for longer duration (from 10 to 50 ms).

We pooled all neurons recorded in different animals, assuming that they represent the same variability of neural responses as an equal number of cells in a single mouse. The activity of the 102 and 130 neurons in anesthetized and awake mouse were organized in 102 and 130-dimensional vectors respectively, containing in each dimension the average firing rate of a recorded cell computed over a certain time bin. Population vectors were built using 8 (on average, anesthetized: 43 ms, awake: 49 ms; Figs. 2 and 3) and 24 bins (on average, anesthetized: 14 ms, awake: 16 ms; similar results were also observed with 14 ms in awake dataset; Fig. 4) per breathing cycle. We recurrently changed the time bin duration for analysis performed in Fig. 5.

Gamma oscillations were also used as a frame to bin the spiking activity and build the population vector. In this case, each M/T cell firing rate was binned with oscillations cycles recorded on the same tetrode. The gamma cycles were then realigned to the average oscillation (19 ms±2; mean±S.D.) over all tetrodes. The realignment was identical than the one used for breathing cycles.

To compute classification performance, one trial per stimulus was chosen to be a test set, and the remaining trials were averaged to be template responses. The Euclidean distances between test trials and all stimuli templates were computed, and trials were assigned to the closest template (i.e. to a stimulus prediction). The percentage of success for odor identity and intensity was the fraction of correct assignments over the total number of assignments. We averaged this percentage over all the odors.

In short, the algorithm creates template vectors for each stimulus based on a fraction of the stimulus repetitions and then assigns each remaining trial to the closest template (i.e. measuring the Euclidean distance).

#### Shuffling of the vector structure

We randomly shuffled each bin containing the firing rate over the breathing cycles for each trial, cell and odor.

#### Construction of cumulative and concatenated vector

The cumulative rate was computed by summing the vectors of each consecutive time bins in the first breathing cycle after the odor onset. At the end of the period of interest (i.e. a complete breathing cycle), the cumulative vector contains the rate summed over all the subsequent time bins contained in the respiratory period (Fig. 5A). The concatenated code consists in concatenating each successive vector to the precedent ones (as an example form the time bin *i*, the vector dimension is *i* multiplied by the number neurons in the population). It keeps the activity history of all the time bins after the odor onset (Fig. 5A).

For the awake dataset, as we used only 5 trials per stimulus, we tested whether the difference in performance observed between the cumulative and the concatenated codes may be due to an undersampling, especially in the case of short time windows. We acquired a second dataset and we used 20 trials per odorant stimulus. We computed the maximum prediction performance for the first cycle after the odor onset while varying the number of trials used trial from 3 to 20 and using different binning window to compute the population vecor. We observed that the prediction performances of the two codes evolved in a similar manner for different number of trials, independently from the time window (Fig. S2). This ruled out the possible bias due to the undersampling.

## Results

### Odorants evoke weak rate change in mitral cells recorded in awake mice

In order to study the potential coding mechanisms in M/T cells underlying odor representation, we recorded ensembles of neurons from the mouse OB in both anesthetized and awake mice (Fig. 2A–B) [22]. The average baseline firing rate across all recorded neurons was 15.4±14.3 (mean±S.D.) and 14.2±14.6 for anesthetized and awake animals (no difference between the distributions, Kolmogorov-Smirnov test, P>0.05), respectively. In anesthetized mice, odorants evoked clear responses in some neurons (∼40% of the cells; see below) as shown in the peri-stimulus time histogram (PSTH) binned over respiratory cycle duration (Fig. 2C). We observed either an increase or decrease of firing rate following the odor onset (see representative examples). In contrast, in awake mice the same set of stimuli evoked weak or no changes in firing rate variations (Fig. 2D). We thus determined whether the recorded neurons responded to the odorants. This was accomplished by measuring in each respiratory cycle, the percentage of cells that significantly changed their firing rate to at least one (out of 7) odorants tested (see Methods). In anesthetized mice a large fraction of M/T cells (∼40%, *n = *102 neurons tested) responded significantly to at least one odorant (Fig. 2E). In contrast, in awake mice the percentage of responding cells was unchanged during odorant application (Fig. 2F; 1.92%±0.44 S.D. and 1.69%±0.64 for baseline and odor periods, respectively; *n = *130 neurons). Thus M/T neurons recorded in awake mice respond much less to odorants by a rate change.

It follows that in awake mice, either a large fraction of M/T cells are not activated by the odorants and carry no information about the stimulus, or the cells respond and encode the stimulus without strongly changing their firing rate (i.e. baseline and odor-evoked rate changes computed over a breathing cycle are not significantly different, Fig. 2F). We explored the ability of neuronal ensembles to encode sensory information. The ensemble activity was quantified using a “population vector” representation, a method (described below) already successfully used for the analysis of olfactory coding [22], [32], [33], [34], [35]. For each neuron, its firing rate was calculated over a specific time window and was fed in individual raw of a vector. Each respiratory cycle was divided into 8 bins of equivalent duration (anesthetised: 50 ms, awake: 40 ms). The temporal evolution of population firing, for each odor trial, is thus described by vector time series. In order to test the actual encoding capabilities of the population activity, we used on a single trial basis, a classification algorithm based on population vector similarity (see Methods). In anesthetized mice, the curve of correct prediction fluctuated between ∼30–60% correct within and across respiratory cycles (Fig. 2G; first cycle: max: 52% and mean: 37±10% S.D.). These values are much greater than chance (14%), confirming that the population activity could be used to decode stimulus information on a single trial basis. In awake mice, the correct prediction was surprisingly (given Fig. 2F) different from chance and even superior to the values obtained in anesthetized animals (Fig. 2H; first cycle: max: 77% and mean: 54±17% S.D.). In summary, a large fraction of M/T cells in awake animals, while rate-invariant over a breathing cycle, still respond to odorants and carry enough information about the stimulus identity to be discriminated by cell ensembles.

### In awake mice, odorants tune the spike timing in the breathing cycle

To encode stimulus information while being rate-invariant, individual neurons must carry information by tuning their spike timing within the respiratory cycle (RC). It therefore follows that only if the bulbar network contains a sufficient population of co-active M/T neurons that such information may be read by higher brain centers. In order to better detect changes in odor-evoked activity, we quantified the number of odorant-responsive cells that contribute to the population code using a method that not only takes into account the firing rate but also redistribution of spike timing in the respiratory cycle. We divided each respiratory cycle into 8 bins of equal duration (40–50 ms). This process permits the represention in time of all averaged odor trials as vector time series of 8 dimensions (i.e. sequence of consecutive RCs). For each neuron, we then computed a principal component analysis (PCA) on all averaged odor trials (see Methods). In the PCA space, baseline and odorant evoked population firing activities appear as points representing individual RCs (Figs. 1 and 3A–B).

To find significant changes in firing patterns after odorant application (Fig. 3), one must take into account the stochastic firing variation in single neurons. To determine which of the odorant responses are significantly different from baseline, on each average trial we used a framework taking into account baseline fluctuations (see Methods and Fig. 1). For cells recorded in anesthetized animals, odor-evoked RC activity was clearly different from the baseline activity (i.e. color points outside the black cloud), a result consistent with the large odorant-evoked rate changes (Fig. 3A). In contrast, in awake mice, the entire set of points (i.e. baseline and odor periods) is more compact though during the odor period, some RCs are clearly spatially located outside the baseline cloud; consistent with the idea that the neurons responded to these odorants (Fig. 3B). To identify those responsive neurons, we determined the respiratory cycles during the odorant application period that were sufficiently far away from the baseline activity to be considered as different and thus responsive. To estimate the number of false responding neurons we also performed the analysis over baseline epochs. We set a standard deviation threshold at which we detect no more than 5% false positive responses over the baseline (Figs 1 and 3C–D). After quantification, 64% of the M/T cells in anesthetized (Fig. 3C) and 29% of the awake animals (Fig. 3D) were found to respond to at least one odorant. We conclude that some neurons recorded in awake animals respond to odorants by tuning the firing activity within the respiratory cycles.

### Efficiency of odor information coding by population of rate-invariant M/T cells

**Figure 6:**
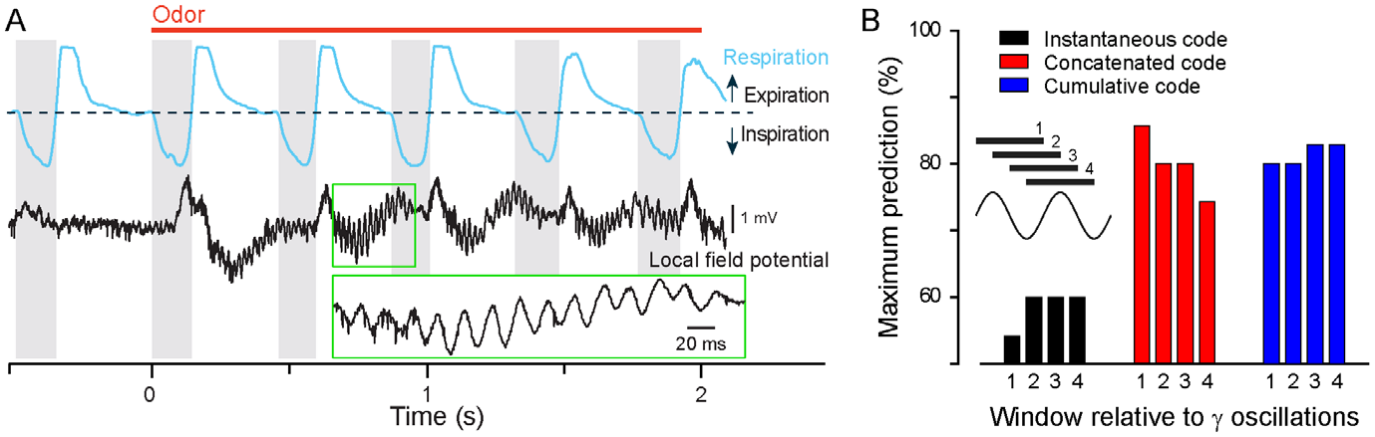
Encoding odorant information in different phases of the gamma oscillations recorded in the OB. (**A**), Traces of the respiration (*blue*) and of the local field potential (*black*) before and during (*red bar*) odorant application. Gamma oscillations are clearly visible in the enlarged part shown in the green rectangle. Light grey boxes indicate inspiration phases. (**B**) Maximum of correct prediction reached in the first respiratory cycle following odor application, computed for the different neural codes (instantaneous, concatenated, cumulative) and plotted as a function of the timing relative to the phase of the gamma oscillation. The prediction curves were computed with population vectors binned over the duration of a gamma oscillation, the beginning of the averaging window varying with the phase of the gamma oscillation (see *schema*).

We next inquired whether the entire population of cells is involved in encoding stimulus information. To address this issue, we constructed a population vector over a reading time window of gamma oscillation duration (16 ms, ∼60 Hz) that we used to compute the correct stimulus prediction curve (Fig. 4). Such oscillations are evoked by odors in the OB (see Fig. 6A) and have been proposed to reflect some sensory processing [10], [36], [37], [38], [39], [40]. For both anesthetised and awake animals, the prediction values were largely above the chance level (Fig. 4A–B). In addition the maximum of prediction decreased in this shorter temporal window when compared to longer time bins duration presented earlier (compare Figs. 2G–H and 4A–B respectively). The population firing activity is thus sufficiently precise to encode odor identity at 60 Hz.

We then computed the correct prediction curve using a subpopulation of 37 out of 102 neurons recorded in anesthetized animals and which excluded the responsive neurons (identified in Fig. 3). As expected, the performance decreased to chance level (Fig. 4C). In awake animals in contrast, after removing the “responsive” cells, the classification performance remained above chance levels (Fig. 4D, maximum: 45%; *n = *92 cells remaining in the population). To eliminate the possibility that this result is due to the difference in the number of cells used in the cellular assemblies, we randomly chose the same number of cells in the population of awake and in the anesthetized animals (i.e *n = *37), and performed the classification with this subset of cells. We performed the random choice process 10 times in order to pick up each time a different subset of cells (Fig. 4E). The average prediction curve was then still markedly greater than chance level (Fig. 4E, max: 40±6%). These results emphasize that cells that are usually neglected due to their weak firing rate changes and considered as non-responsive can still contribute to the neural code, as revealed by the population analysis.

In awake mice, rate-invariant cells contain sufficient information to correctly encode odorant information as cell assemblies. The discharge patterns of the population found at each trial are sufficiently robust to predict the stimuli identity. Therefore each cell may be precisely time-locked (at least across 16 ms windows), leading to a typical odorant-evoked ensemble pattern. To test the importance of the discharge timing, we changed, inside individual breathing cycle, the spike pattern structure for all recorded neurons. For that, we randomly shuffled the 16 ms time bins inside each breathing cycle for each trial, odor and cell. With such shuffled population, the ability to predict stimulus identity has vanished (classification performance is now at chance level; Fig. 4F).

Taken together, these data indicate that precise timing of the firing in the neuronal population is critical to encode odorant identity accurately.

### Impact of the reading time window duration on different decoding mechanisms

The preceeding findings highlighted the importance of temporal information to encode odorant identity by cell assemblies. However, to understand the time scale at which the population activity yields the highest prediction and to assess which coding scheme (for example: cumulative rate or temporal sequence) would be more predictive for different readout window durations we have done the following analysis. Since animals are able to make a decision about odorant identity in a single sniff (∼200 ms/ [41], [42], [43], [44], [45], [46]), we performed the analysis on the first breathing cycle and we varied parametrically the duration of the analysis window. We computed the stimulus prediction curve either in individual time bin (instantaneous) or by summing (cumulative) or concatenating (concatenated) the bins over time. The instantaneous readout would be similar to a rate snapshot reading, in which each point is independently informative. In contrast, reading information over time takes into account past history. We considered two models: either looking at spike accumulation over time (cumulative rate) or at the information contained in the activity sequence (i.e. temporal features) by performing the concatenation of each population vectors of subsequent time bins. The latest description preserves the information contained in fine temporal details of activity, which is lost when taking the average of the vectors.

In individual time bins (instantaneous readout), correct odor prediction increased upon enlarging the time window duration, reaching a maximum of ∼80% for bins of 100 ms and then further decreasing (Fig. 5A–B). For windows smaller than 50 ms, prediction decreases until reaching a value comparable to baseline at time scales close to the spike timing (3 and 5 ms) (Fig. 5B). In contrast, when the evolving history of spiking activity over time (cumulative or concatenation) is taken into consideration the information can be extracted at these time scales reaching up to 80% prediction (Fig. 5A–B). We observed, however, a notable difference between the cumulative rate and the concatenated codes. The maximum prediction reached in the breathing cycle for the cumulative rate analysis remains stable for all time bin durations <100 ms, whereas the concatenated model shows a decreasing prediction for short time windows (Fig. 5A upper left panel and Fig. 5B). Interestingly, for windows <10 ms, the prediction decreases and becomes lower than the cumulative rate code (Fig. 5B). Therefore, over the respiratory cycle, the maximum prediction of the cumulative rate code is more resistant to highly fluctuating noise present at a fast time scale but is less predictive than the concatenated code at time scales longer than 100 ms.

Moreover, the prediction curve evolves differently during the respiratory cycle for both codes, a parameter that could be of behavioral importance when considering the observed speed accuracy tradeoff in odor discrimination experiments [42], [43]. The concatenated model reaches faster the maximum prediction for specific time windows (for example 12 ms; Fig. 5A upper-right panel) than does the cumulative model. Hence, we quantified this difference in performance by plotting the ratio between the prediction curves of the two codes for different time bin duration (Fig. 5C). We computed this analysis for three parts of the breathing cycle (from odor onset to either 100 ms, 200 ms or the end of the breathing cycle). In all cases, the cumulative rate code was a better predictor at scales close to the spike timing but became less efficient for time windows between 6 to 50 ms (Fig. 5C). We also observed that the ratio increased when earlier part of the breathing cycle was considered, reflecting that concatenated code prediction reaches higher performances directly after odor onset. At fast time scales (<6 ms), cumulative rate is better in encoding odorant information whereas the concatenated code reaches high predictive values more rapidly (for reading windows between 6 and 50 ms). In order to achieve a trade-off between accurate and fast discrimination, these results highlight the existence of an optimal reading time window having a duration of 10 to 50 ms, in which the firing activity may be analyzed. We then inquired as to the nature of an internal clock that could be used to chop up the spike sequences to maximize the reading of the sensory information.

Interestingly, the optimal time window for odorant discrimination in the olfactory bulb is compatible with the duration of a single gamma oscillation cycle. As such, the gamma oscillations could be used as an internal clock used to convey the spikes emmited by the M/T cells. To test this hypothesis, we detected the gamma cycles from our recordings (Fig. 6A) and we computed population vectors using the gamma oscillations duration as a reference. Across trials, the gamma cycles were realigned to each other (similar to the breathing cycle alignment, see Methods), the average duration of a gamma cycle being 19 ms±2 (mean±S.D.). In addition, we used different parts of the gamma oscillations as a starting point to generate different population vectors: the peak-to-peak period, the first inflexion point, the trough-to-trough period and the second inflexion point (Fig. 6B). No matter how the information had been parcelled, we observed high performances of prediction for the concatenated and the cumulative codes. It confirmed that the gamma oscillation could act as a framework conveying sufficient information to discriminate between different stimuli. In addition, the choice of the time reference was important in order to retrieve the maximum amount of information for the concatenated code. Indeed, we observed that this code is very sensitive to the gamma phase chosen to compute the population vector (Fig. 6B). The part of the gamma cycle being the most informative, starts at the rising phase of a gamma oscillation. These results indicate that information about odorant identitiy can be parcelled by gamma oscillations cycles, each of which may act as an embedding structure for the spikes.

In conclusion, these data suggest that odorant information retrieved in the population activity is read as spike packets (i.e. temporal blocks) of a specific duration to reach an optimal trade-off between fast and accurate odorant identification.

## Discussion

In this study, we analyzed how odorant information is processed in population of M/T cells. In contrast to anesthetized animals, where rate changes dominate, we showed that a large fraction of OB M/T cells are rate-invariant over a breathing cycle and respond to odorants in awake mice mainly by changing the timing of the spikes in respect to the breathing cycle. We found that these rate-invariant neurons transmit sensory information as co-activated cell population in different time windows. In awake animals, sensory information may be decoded as spike packets sequences by downstream network in order to reach a tradeoff between rapid and accurate odor discrimination. We propose that OB gamma oscillations act as an internal clock to drive the spikes packets sequences.

### M/T cell responsiveness to odorants in awake mice

Numerous studies in anesthetized mice report a large percentage of M/T cells responded to odorants by changes in their firing rate [20], [21], [22], [23], [24], [25]. In awake animals, we confirmed, at a larger population level, an overall reduction of odorant-evoked rate change [47]. In this dataset, no M/T cells exhibited a significant odorant-evoked rate change (Fig. 2 and 4) but they respond to odorants by redistributing their spikes within the cycle, as described recently for some neurons [32], [48].

Why were the cells in awake animals not found to significantly respond by a rate change in an averaged respiratory cycle? Considering shorter time bins, it is possible that some cells display a transient rate change in a respiratory cycle phase that would be averaged out when considering the mean cycle discharge. However, it is difficult to statistically extract such period on a small number of trials (five for awake mice) and without considering the entire cycle discharge as firing fluctuate substantially in different part of the cycle (even in the baseline). Neurons may still display weak and transient firing rate changes but below the intrinsic fluctuations of the baseline firing. At a population level, these small rate adjustments could still be detected and used by downstream brain centers as shown in our analysis by the ability to predict sensory information using the rate codes for long analysis window duration spaning the inspiration.

Importantly, we don't claim that none of M/T cells change their firing rate during any odorant presentation as could be observed previously for some neurons [32], [48]. In this dataset, we did not observe strong firing changes, which may be due to different reasons. First, the M/T cells recorded in different part of the OB may exhibit different response profiles (we recorded mainly cells in the dorsal part). Second, the number of repetitions per odorant stimulus may be another difference. In our study, we used only few trials per odorant whereas in other reports, several hundred trials were used and sometimes during behavioral tasks. While having a small number of trials may underestimate the number of cells significantly changing their firing rate, increasing the number of trials may also induce some forms of short term plasticity [49]. In addition, the response of M/T cells in animals engaged in a behavioral task may be modified by neuromodulatory centers [50]. Finally, the concentrations and the nature of the odorants used (monomolecular vs. mixtures) may be another source of variability. Indeed, we observed some neurons displaying firing rate changes when using monomolecular odorants at higher concentrations (data not shown).

In summary, M/T cells may exhibit complex behaviors following odorant presentation. While some neurons can exhibit obvious rate changes, a large fraction of the population can still respond to the stimuli by exhibiting change in their spike timing inside the respiratory cycle. In conclusion, while many M/T cells in awake animals have previously been considered as non responsive and sparse by rate analysis, in fact, they respond to odorants by change in temporal patterns, contributing to sensory coding.

### Implication for coding

M/T cells have been proposed to encode odors by strongly changing their firing rate albeit to only few odorants, implying that M/T cells are narrowly tuned and that the odor code is sparse [21], [23], [25], [47]. However, these studies do not take into consideration rate invariant neurons, commonly thought to be unresponsive and thus not contain information regarding the odorant identity. We showed that ensembles of rate invariant neurons can encode odorant identity with high accuracy. Indeed, a striking finding was that, the population of “unresponsive” cells (∼70%) contributed to the neural code as the ensemble activity could be used to predict the stimuli presented. Therefore, analysis performed at a single cell level underestimates the percentage of cells possibly contributing to sensory information transfer. This result highlights that inferring the neural code based on single cell response profile tends to give only a partial view of how sensory information is processed.

While individual neurons don't change their global firing rate, how may the OB network encode sensory information? One strategy is to encode sensory information by increasing synchrony [3], [4], [5]. Theoretical studies showed that neurons can encode sensory information by synchronizing their spikes without significantly changing their firing rate [2], [30], [31]. Experimental studies in the insect olfactory system support the role of synchrony in odor coding, though neurons that display strong rate changes also contribute to the odor code [3], [4], [5]. In our study we couldn't precisely assess synchrony between neurons at the time scale of individual spike as we used a limited number of trials per stimulus. Future experiments using a larger number of trials would be needed to precisely evaluate the influence of synchrony in different part of the cycle to encode odorant information. Although synchrony between spikes may still be important, we propose a slightly different mechanism of coactivation. We suggest that M/T cells act as cell assemblies carrying odorant information by finely tuning subsequent spiking segments through the breathing cycle. As a consequence, decoding performance achieved by downstream network would be strongly influenced by the duration of the read out time window.

### Specific read-out time window for temporal code

How may downstream networks such as the piriform cortex be reading incoming inputs? As in other systems [15], [16], the decoder may need a reference to integrate sensory information. Obviously an essential reference is the start of the sensory stimulation but additional reference time points may be needed by the decoding networks to maximize information readout. Here, we defined these optimal readout window durations for different coding strategies. The instantaneous firing rate code led to decreasing prediction performances for short duration analysis windows (<50 ms), reaching the level of the baseline noise (for 4 ms). By computing a cumulative spike count or a concatenated vector (temporal sequence), we showed however that both coding strategies led to higher prediction of odorant identity at faster time scale than the instantaneous rate code. We further report that the concatenated code performs better in the early phase of the respiratory cycle, corresponding to the inhalation phase. This result is in agreement with a recent report, which has shown that a concatenated model is more efficient than a rate model to encode odorantss in a read out window of 20–40 ms in rats trained to a two choices discrimination task [32]. Our study adds two important observations to their results. First, in untrained mice we reached high prediction level by using populations of rate invariant neurons and using few odorant applications (5 trials versus several hundreds per stimulus), This situation approaches more natural olfactory behavior where an odor is recognized by naïve animals, avoiding possible remodelling of activity after behavioral training paradigms and repetitive odorant applications [49]. Second, we showed a fast decrease in performance of the concatenated model close to the spike count time scale (<6 ms), a time window that was not assessed in the latter study. This coding scheme eventually becomes too much sensitive to noise and decreases drastically its prediction performance, while the cumulative code remains stable and reaches a high performance level. Beyond the boundaries of this window, the instantaneous rate or the cumulative rate codes perform better than the concatenated code. However, none of them reaches a maximum level of performance as fast as the concatenated code in the optimal read-out time window during the inhalation phase (80–100 ms).

Therefore, the olfactory cortex may decode the OB outputs as subsequent spikes trains embedded in ∼12–50 ms windows. This particular read out scale may allow the system to reach an optimal trade-off between accuracy and speed of odor categorization, as observed during behavior [43]. Interestingly, coincident convergence of synaptic inputs from M/T cells of different glomerular units onto piriform cortex pyramidal cells efficiently triggers spiking for this particular duration [51], [52].

Therefore, to obtain an optimal trade-off between discrimination accuracy and speed as observed during behavior [43], the olfactory cortex may decode the information coming from the olfactory bulb in subsequent time segments of ∼12–50 ms, keeping the information of precedent packets. Interestingly, coincident convergence of synaptic inputs from M/T cells of different glomerular units onto piriform cortex pyramidal cells efficiently triggers spiking for this particular duration [51], [52].

Moreover, the duration of the optimal readout time window corresponds either to the duration of a gamma oscillation cycle or to the duration of a fast beta oscillations cycle. Gamma oscillations are commonly observed in the OB and has been proposed to be essential for odor processing [10], [36], [37], [38], [40], [53], [54], [55]. Thus, it can represent a driver for the temporal spike packets. *In vitro*, gamma oscillations enhance the spike timing reliability, increasing discrimination between odors. The rising phase of the oscillation seems to drive spiking activity and optimize the stimulus information content [10], [36], [37], [38], [40], [53], [54], [55]. Our results provide a similar view and show that gamma oscillation can play the role of the internal reference to drive the spiking segments in the neuronal population. Moreover, we also observed that varying the internal reference defined by gamma oscillation phase change also the information content of the spiking trains. Thus, rising phase of the gamma cycle led to the maximum odor discrimination when using the concatenated code.

Recent work in the piriform cortex has shown that pyramidal neurons are phase-locked to beta oscillations of the local field potential recorded in the cortex [56]. Interestingly, different cortical neurons prefer to spike at different phases of the LFP beta oscillation. It is possible that the variability of the preferred phase may correspond to the ability of different cortical neurons to integrate spiking packets locked to different OB gamma oscillation cycles. Anyhow, the way M/T cell spikes are conveyed to the piriform cortex and integrated in a broader frequency range still need to be fully understood.

In conclusion, we showed that a large fraction of M/T cells may encode odorants without changing their global firing rate. These cells form assemblies of temporally coactive neurons. We propose that sensory information carried by these assemblies is embedded in the temporal spiking patterns that may not be processed by the cortex independently but in spike segments of specific duration. The gamma oscillations may represent an advantageous reference time frame to drive the spikes [16] in subsequent segments. The information would be optimally decoded from the total sequence of segments over the inspiration period, providing a trade-off between fast and accurate discrimination.

## Supporting Information

Figure S1 **Schema of the breathing cycles realignment across trials.** (**A**) Schema of the breathing cycle after odorant application onset for three different trials. The grey boxes represent the inspiration phase. The duration of the respiratory cycles can change from trial to trial (but also within the same trial). M/T cell spikes are indicated in blue. (**B**) Schema of the realigned breathing cycles. The inspiration onset from each trial is realigned to the mean breathing duration (393±15 S.D.). The longer breathing cycles were then cutted. The shorter were prolonged. It is noteworthy that the spike timing relative to respiration cycle onset is not changed by this procedure.
(TIF)

Figure S2 **Dependence of the classification analysis on the number of trials per stimulus.** We tested such dependence for both neural codes (concatenated and cumulative) and for several analysis window durations. We always observed similar curves for both codes, independently of the binning duration used to compute the population vector. All predictions have been normalized to the prediction reached for 20 trials.
(TIF)
