# Supplementary figures and images for: Encoding Odorant Identity by Spiking Packets of Rate-Invariant Neurons in Awake Mice
Source: PLoS One. 2012 Jan 17;7(1):e30155. doi: 10.1371/journal.pone.0030155 (PMC3260228; doi:10.1371/journal.pone.0030155)

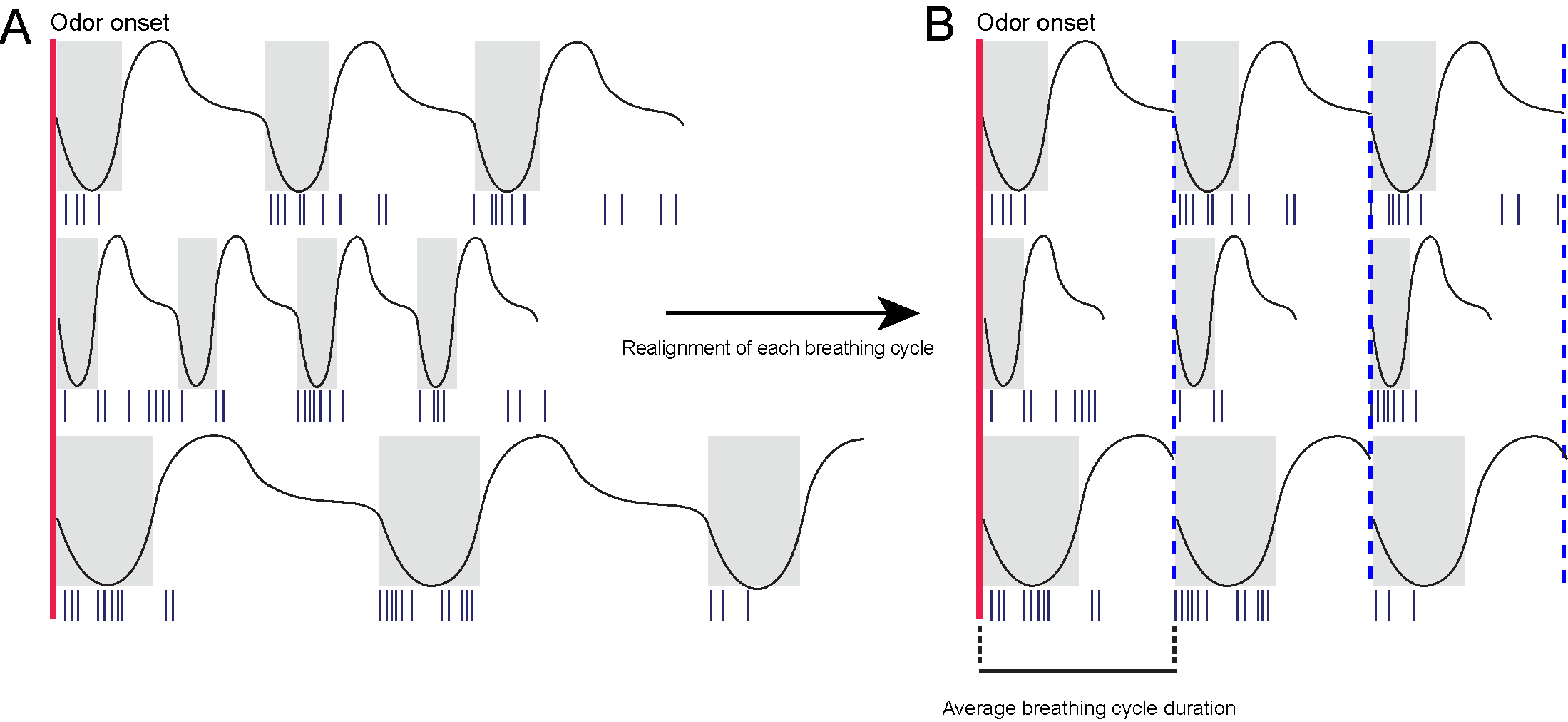

Supplement: Figure S1 — Schema of the breathing cycles realignment across trials. (A) Schema of the breathing cycle after odorant application onset for three different trials. The grey boxes represent the inspiration phase. The duration of the respiratory cycles can change from trial to trial (but also within the same trial). M/T cell spikes are indicated in blue. (B) Schema of the realigned breathing cycles. The inspiration onset from each trial is realigned to the mean breathing duration (393±15 S.D.). The longer breathing cycles were then cutted. The shorter were prolonged. It is noteworthy that the spike timing relative to respiration cycle onset is not changed by this procedure. (TIF) [file pone.0030155.s001.tif]

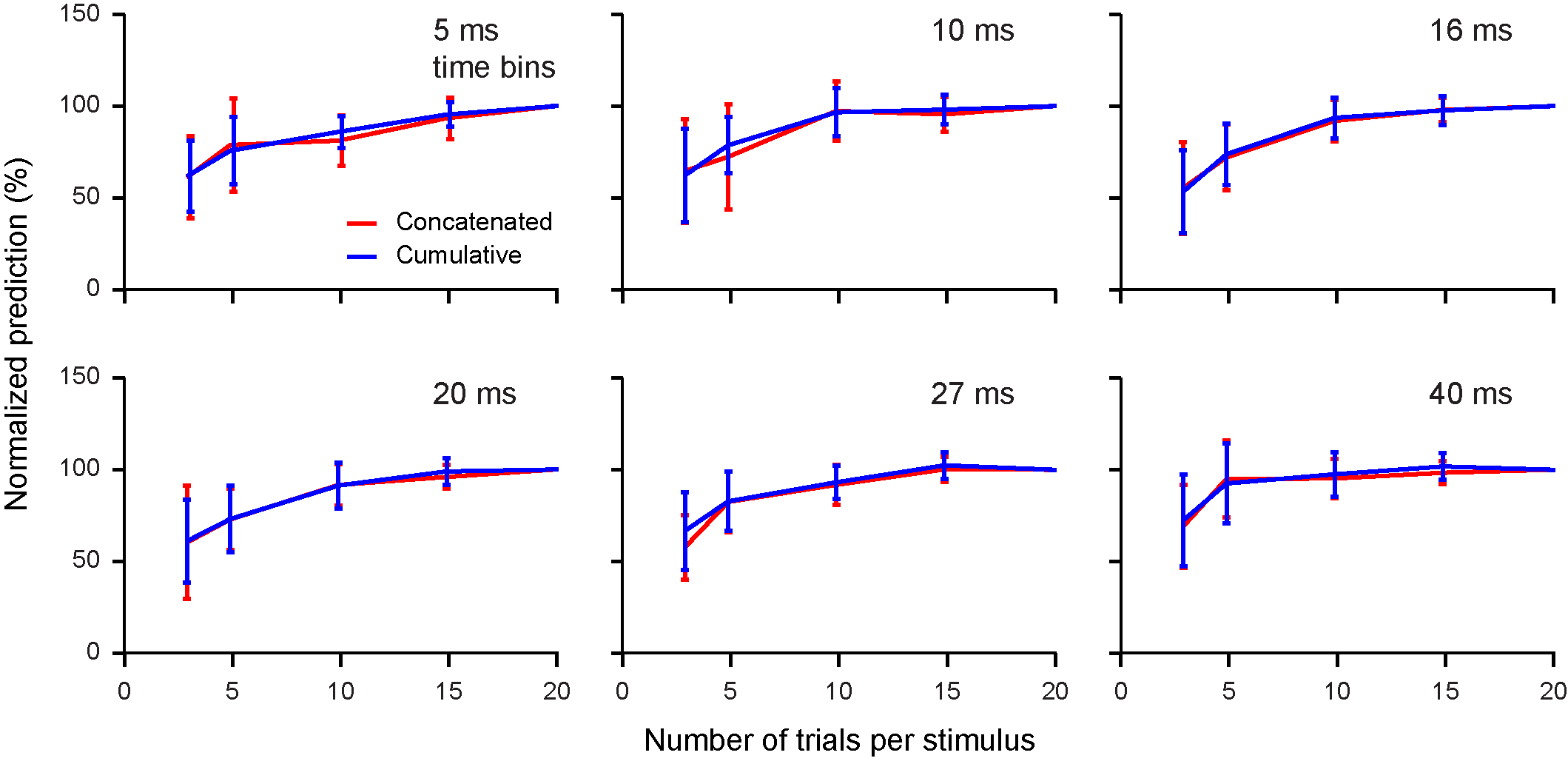

Supplement: Figure S2 — Dependence of the classification analysis on the number of trials per stimulus. We tested such dependence for both neural codes (concatenated and cumulative) and for several analysis window durations. We always observed similar curves for both codes, independently of the binning duration used to compute the population vector. All predictions have been normalized to the prediction reached for 20 trials. (TIF) [file pone.0030155.s002.tif]
